# Variable selection methods for predicting clinical outcomes following allogeneic hematopoietic cell transplantation

**DOI:** 10.1038/s41598-021-82562-0

**Published:** 2021-02-05

**Authors:** Chloé Pasin, Ryan H. Moy, Ran Reshef, Andrew J. Yates

**Affiliations:** 1 Department of Pathology and Cell Biology, Columbia University Irving Medical Center, New York, NY 10032 USA; 2 Department of Medicine, Memorial Sloan Kettering Cancer Center, New York, NY 10065 USA; 3 Columbia Center for Translational Immunology and Division of Hematology and Oncology, Columbia University Irving Medical Center, New York, NY 10032 USA

**Keywords:** Allotransplantation, Statistics, Predictive markers

## Abstract

Allogeneic hematopoietic cell transplantation (allo-HCT) is a potentially curative procedure for a large number of diseases. However, the greatest barriers to the success of allo-HCT are relapse and graft-versus-host-disease (GVHD). Many studies have examined the reconstitution of the immune system after allo-HCT and searched for factors associated with clinical outcome. Serum biomarkers have also been studied to predict the incidence and prognosis of GVHD. However, the use of multiparametric immunophenotyping has been less extensively explored: studies usually focus on preselected and predefined cell phenotypes and so do not fully exploit the richness of flow cytometry data. Here we aimed to identify cell phenotypes present 30 days after allo-HCT that are associated with clinical outcomes in 37 patients participating in a trial relating to the prevention of GVHD, derived from 82 flow cytometry markers and 13 clinical variables. To do this we applied variable selection methods in a competing risks modeling framework, and identified specific subsets of T, B, and NK cells associated with relapse. Our study demonstrates the value of variable selection methods for mining rich, high dimensional clinical data and identifying potentially unexplored cell subpopulations of interest.

## Introduction

Allogeneic hematopoietic cell transplantation (allo-HCT) is used as a curative treatment for many blood cancers and non-malignant conditions. It involves transferring hematopoietic cells, including stem cells, from a healthy donor to induce a complete or partial replacement of the recipient’s hematopoietic system. The success of the procedure relies on the effective reconstitution of the immune system and the eradication of tumor cells by the donor cells (the graft-versus-tumor effect)^1^. However, the success of allo-HCT can be compromised by a number of clinical events: opportunistic infections, relapse, or graft-versus-host-disease (GVHD). GVHD occurs when cells from the graft recognize minor histocompatibility antigens expressed on non-hematopoietic cells, and cause damage in tissues—typically gut, liver, and skin^2^. There are two forms; acute (aGVHD) or chronic (cGVHD). Historically, the differential diagnosis is made based on the time since allo-HCT (before or after 100 days), but recent recommendations classify GVHD by its clinical manifestation^3^. Preclinical models and clinical studies have advanced our understanding of the pathogenesis of GVHD^4^ and identified factors influencing its risk of occurrence, such as the genetic distance between donor and recipient (unrelated versus sibling), conditioning intensity^5^, and cytomegalovirus (CMV) seropositivity^6^. Graft source, patient age, and the donor/recipient gender combination are also some of the factors included in the European Bone and Marrow Transplant group risk score, which is used to predict HCT outcomes^7^. Events soon after transplant may set the stage for later outcomes, and identification of immunological variables associated with phenotypes could help in understanding GVHD pathogenesis^8^.

Advances in biotechnology, and in particular methods based on single-cell characterization such as flow or mass cytometry, have given many insights into the trajectories of the immune response to self and tumor antigens following allo-HCT^9–11^ and led to the identification of associations between some cell subsets and clinical outcomes^12^. For example, the risk of aGVHD was increased in patients with lower CD56$^{bright}$ NK cells within two months after allo-HCT^13^ and NK cell numbers 30 days after HCT were also negatively associated with the risk of aGVHD and death^14^. Studies focusing on T cells revealed that higher numbers of CD38$^{bright}$ effector memory CD8$^+$ T cells^15^ and lower frequencies of regulatory T cells within CD4$^+$ T cells^16^ were predictive of the occurrence of aGVHD. Further, aGVHD and cGVHD patients were found to exhibit lower numbers of $\hbox {IgM}^+$ memory B cells^17^, and some evidence of B cell exhaustion was found in cGVHD patients^18^. In addition, levels of CXCL9 100 days after transplant and levels of CXCL10 measured pre-transplant were found, in separate studies, to predict cGVHD up to 1 year^19,20^. Importantly, variables measured 30 days after transplant can also predict clinical outcome: multiple biomarkers are predictive of non-relapse mortality up to 12 months post-transplant^21^ and donor chimerism levels predict relapse and overall survival^22^. However, most studies aiming to predict a patient’s clinical evolution focus on preselected and predefined cell subsets, and do not explore the full potential of multiparametric immunophenotyping, which can enumerate tens or even hundreds of phenotypes at once.

Here, we perform unbiased identification of clinical and immunological variables (specifically, cell subpopulations present 30 days after allo-HCT) that are associated with 3 clinical outcomes experienced by allo-HCT recipients: relapse, acute GVHD grade 2 to 4 (aGVHD24), and chronic GVHD (cGVHD). We use sophisticated and complementary statistical tools to analyze flow cytometry data and select relevant cell subpopulations. We do this within a competing risk framework, studying the time to the first event experienced by patients following allo-HCT. Competing risk models are widely used in transplant studies, and are usually implemented to study the effect of a small number of clinical factors on outcome. Here we examine a dataset in which the number of potential covariates of interest far exceeds the number of patients, a scenario that has become ubiquitous as high-throughput assays are now widely used in the biomedical sciences. To deal with these high-dimensional data we explore variable selection methods, which are well-developed in the context of classification or regression, but have only recently been adapted to the setting of competing risk analysis^23–25^.

## Results

### Patient characteristics

**Figure 1:**
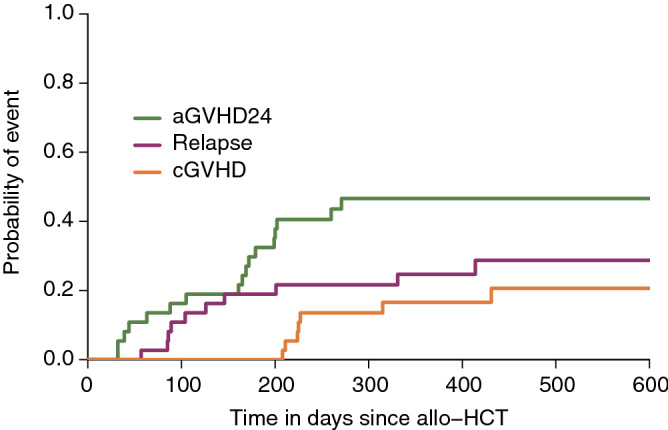
Probabilities of occurrence of each event over time in the studied sample.

We studied data generated during a previously reported phase 1/2 clinical trial^26,27^, which assessed the safety and efficacy of the addition of a CCR5 antagonist (maraviroc) to standard GVHD prophylaxis. Immunophenotyping was performed by flow cytometry 30, 60 and 90 days after allo-HCT on a subset of patients in the trial. Given the small number of measures at days 60 and 90, we focused on a subset of 37 patients for which the measures of 82 cell subpopulations at day 30 were complete. Their clinical characteristics are detailed in Table 1, and the cell subpopulations are listed in Supplementary Text S1 online. Among these patients, 17 experienced aGVHD24 as a first event after allo-HCT, 10 relapsed and 8 experienced cGVHD. Two patients received a donor lymphocyte infusion (DLI) before experiencing any of these three events. Figure 1 summarizes the raw time-to-event data, yielding the estimated probabilities of occurrence of each event over time in the study sample.

The cell subset frequency data are summarized in Supplementary Figure S1 online and available in full in Supplementary Data S1 online.

**Table 1:** Characteristics of the subset of patients used in the analysis. *DLI* Donor lymphocyte infusion.

	Controls	Maraviroc	Total
	n=19	n=18	n=37
Donor age, mean (sd), years	42 (17)	40 (15)	41 (16)
Recipient age, mean (sd), years	59 (10)	61 (6.9)	60 (8.5)
**Recipient sex, n**			
Male	14	12	26
Female	5	6	11
**Cytomegalovirus status**, n			
Recipient positive	9	5	14
Donor positive	7	7	14
**Diagnosis, n**			
Acute Myeloid Leukemia	9	8	17
Myelodysplastic Syndrome	4	4	8
Other	6	6	12
**Donor matching, n**			
Matched related donor	8	6	14
Matched unrelated donor	11	12	23
**Time to neutrophil engraftment, median [range], days**	14.5 [10;23]	17 [7;27]	16 [7;27]
**Time to platelet engraftment, median [range], days**	17 [5;35]	22 [11;84]	18 [5;84]
**Number of infections prior to day 100**			
0	13	12	25
One or more (1, 2 or 3)	6	6	12
**First event experienced, n**			
aGVHD24	13	4	17
cGVHD	3	5	8
Relapse	3	7	10
Censored by DLI	0	2	2
**Time to first event, median [range], days**			
aGVHD24	169 [32;271]	84 [32;202]	165 [32;271]
cGVHD	431 [227;1415]	224 [208;315]	226 [208;1415]
Relapse	89 [57;414]	126 [85;331]	115 [57;414]

### Immunological and clinical factors associated with clinical outcome

We aimed to identify cellular phenotypes present 30 days after allo-HCT that were associated with any of the three clinical outcomes under consideration. To do this we performed variable selection within a competing risks framework. We sought to identify variables associated with either the *cause-specific hazard* (CSH), which is the instantaneous rate of occurrence of a given event among the patients still event-free; or the *subdistribution hazard* (SH), from which one can derive the cumulative incidence function (CIF). This yields the probability of occurrence of a given event over time, in the presence of competing events. The CSH and the SH approaches provide complementary information. In practice, variables associated with the CSH give insights into the mechanisms inducing that event: the corresponding hazard ratio quantifies the impact of the covariate on the event without considering the effect of competing events. On the other hand, variables associated with CIF are more relevant for prognostic research as they can be used in clinical prediction models and the development of risk scores^28–32^. A side-by-side analysis of the CSH and SH is recommended in order to achieve a complete understanding of the event dynamics^33^.

Theoretical and computational details are given in the Methods section.

#### Cause-specific hazard model

We assumed that the effects of covariates on the time-dependent CSH of each event could be described with a Cox model^34^. We present our results as time-independent hazard ratios (HR), which measure the effect size of a covariate from the vector $Z = (Z_1, \dots Z_m)$ of all covariates—if covariate $Z_p$ is continuous, the HR is the relative change in the CSH for event *k*, at any time *t*, between two imaginary patients *j*,$j'$ still event-free, who differ only in the covariate $Z_{p,j'}=Z_{p,j}+1$. If $Z_p$ is binary, the HR is the change in the CSH between the two categories, with all other variables held constant. We performed variable selection on the CSH using elastic-net penalization (see Methods, and ref.^35^). Table 2 summarizes the models obtained for each event, as selected by the Bayesian information criterion (BIC)^36^, and the corresponding HR estimates.

There were 4 patients whose neutrophil count remained > 500/$\mu$L and/or whose platelet count remained > 20K/$\mu$L. For these individuals, the time to engraftment could not be properly defined. We first analyzed all 37 patients, excluding these two variables. We then analyzed the subset of 33 patients with complete data; time to neutrophil engraftment and time to platelet engraftment were transformed into binary variables ($\le$ or >15 days for time to neutrophil engraftment; $\le$ or >20 days for time to platelet engraftment). Adding these variables did not improve on any of the final models selected. Therefore, the analyses presented below were performed on the the full set of 37 patients, excluding these two engraftment measures.

Our analysis showed that treatment with maraviroc decreased the instantaneous risk of developing aGVHD24 by 77% (HR = 0.23, 95% CI 0.07–0.80) and had no significant association with relapse or cGVHD. A higher instantaneous risk of aGVHD24 was also associated with lower numbers of effector memory CD8$^+$ T cells expressing the chemokine receptor CCR5 (HR = 0.67, 95% CI 0.52–0.86), and higher numbers of naive CD4$^+$ T cells expressing CCR5 (HR = 1.39, 95% CI 1.08–1.80), suggesting a higher inflammatory activation status in circulating GVHD-causing naive T cells, that generally do not express CCR5 at steady state. This finding was independent of receiving maraviroc: the model without interactions between the cell subsets was favoured over the model with no interactions ($\Delta$BIC = 4.6). Additionally, there was no statistical difference in the frequencies of effector memory CD8$^+$ T cells and naive CD4$^+$ T cells expressing the chemokine receptor CCR5 between patients receiving maraviroc and the controls (*t* test, *p*= 0.25 and 0.33 respectively). Following estimation by cross-validation, we found that the time-dependent AUC of the selected model was higher than the AUC from a model containing only the maraviroc treatment variable, although confidence intervals were overlapping; at $t=100$, the AUC of the selected model was 0.74 (0.47–0.96) versus 0.63 (0.28–0.84) for the model with maraviroc treatment only, and at $t=200$, these measures were 0.76 (0.50–0.88) versus 0.72 (0.52–0.84). Although the selected model was clearly favoured using the BIC, no individual marker was significantly associated with the instantaneous risk of cGVHD at the 0.05 threshold, reflecting the relatively small size of the patient sample.

A 10-year increase in donor age substantially increased the cause-specific hazard of relapse (HR = 4.46 (95% CI 1.66–12.0)). We also identified some cellular phenotypic associations. A higher instantaneous risk of relapse was associated with lower numbers of CD16$^\text {hi}$ NK cells and differentiated effector memory (CD27$^-$CD28$^-$) CD8$^+$ T cells (HR = 0.24, 0.07–0.83), and HR = 0.58, 0.37–0.89, respectively). Relapse was also associated with higher numbers of memory (CD27$^+$ $\hbox {IgD}^-$) B cells (HR = 2.87, 1.13–7.32) and CCR5$\text {+}$ effector memory CD8$^+$ T cells, although the latter only in the patients receiving maraviroc treatment (HR = 1.73, 1.08–2.78). The model with an interaction between CCR5$\text {+}$ effector memory CD8$^+$ T cells and maraviroc variables had marginally greater support than the model without the interaction ($\Delta$BIC = 2.2).

**Table 2:** Variable selected in final CSHMs for aGVHD24, relapse and cGVHD, with their hazard ratios (HRs), confidence intervals (CI) and *p* values. All models were adjusted on the treatment variable (maraviroc).

Variable	HR	95% CI	*p* value
**aGVHD24**			
CD8$^+$ EM CCR5$^+$	0.70	(0.56–0.89)	0.003
CD4$^+$ Naive CCR5$^+$	1.37	(1.06–1.77)	0.02
Maraviroc	0.23	(0.067–0.78)	0.02
**cGVHD**			
CD4$^+$ EMRA CCR5$^+$	2.02	(0.93–4.39)	0.08
Recipient sex (ref=male)	0.09	(0.008–1.2)	0.07
Maraviroc	2.92	(0.24–36)	0.4
**Relapse**			
B cell CD27$^+$ $\hbox {IgD}^-$	2.87	(1.13–7.32)	0.03
NK CD16$^\text {hi}$	0.24	(0.067–0.83)	0.02
CD8$^+$ EM CD27$^-$CD28$^-$	0.58	(0.37–0.89)	0.01
CD8$^+$ EM CCR5$^+$ in maraviroc recipients	1.73	(1.08–1.28)	0.02
Age donor	1.16	(1.05–1.28)	0.02
Maraviroc	1.20	(0.17–8.2)	0.9

#### Subdistribution hazard model

We performed variable selection on the subdistribution hazards (SH) using a likelihood-based boosting approach (see Methods, and ref. ^23^). The SH model was initially defined by Fine and Gray^37^ to allow a direct interpretation of the effects of variables on the probability of an event. The model assumes that the subdistribution hazards follow a Cox model. Although the formulation of the SH model is similar to that of the CSH model, a subdistribution hazard ratio (SHR) cannot be interpreted as an epidemiological HR or a modification of an apparent risk. A time-independent SHR for covariate *p* corresponds only to the change in the SH between two hypothetical patients *j*,$j'$ still event-free and with with identical covariates except for covariate *p*, with $Z_{p,j'}=Z_{p,j}+1$. The signs of the selected variables’ coefficients ($\beta _{k,p}=\hbox {log}$(HR) < or > 0, equivalent to a HR < or > 1) indicate the direction of their effect the probability of outcome, but their absolute values HR do not have a straightforward interpretation^28–30^. However, the advantage of the SH model compared to the CSH model is that a variable having a statistically significant effect on the SHR has also a statistically significant effect on the CIF, which is not the case in the CSH model^38^. Additionally, variables found to be associated with a clinical outcome in the SH model can be used to develop individual prognostic scores^32^, which can assist with clinical decisions such as treatment adaptation.

**Figure 2:**
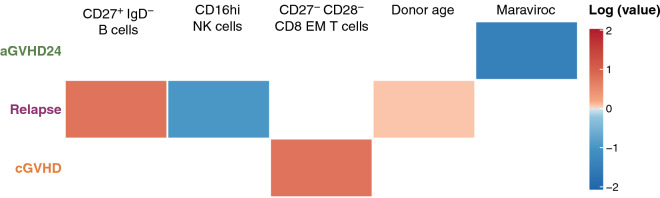
Selected variables and their coefficients (the logarithm of the subdistribution hazard ratio). Positive coefficients (red) are associated with increased event probabilities; negative coefficients (blue) with decreased probabilities.

In Fig. 2, we present the variables selected for each clinical outcome and their associated coefficients. We found that higher numbers of CD27$^+$ $\hbox {IgD}^-$ memory B cells and increased donor age increased the probability of relapse (SHR = 2.19, 95% CI 1.18–4.06 and SHR = 1.09, 95% CI 1.03–1.16 respectively). The role of B cells in the early recovery phase after transplant has not been extensively studied but murine studies show that B cells participate in alloantigen presentation, thus have a role in both the graft-versus-tumor and graft-versus-host responses^39^. CD16$^\text {hi}$ NK cells were negatively associated with relapse (SHR = 0.39, 0.20–0.77) which supports an important role of CD16$^\text {hi}$ NK cells (a mature and highly cytotoxic subset) in the graft-versus-tumor response, which has been previously suggested in human studies^40^. As with the CSH model, we found that the time-dependent AUC of the selected model was higher that the AUC from a model containing only the maraviroc treatment variable, but confidence intervals overlapped; at $t=100$, the selected model’s AUC was 0.78 (0.43–1.0) versus 0.60 (0.36–0.80); and at $t=200$, 0.84 (0.58–1.0) versus 0.72 (0.56–0.84). We also found that patients treated with maraviroc were less likely to experience aGVHD24 than controls, consistent with previous studies^41^. We estimated a SHR of 0.25 (0.08–0.78), considering relapse and cGVHD as competing risks; the earlier point estimate of 0.42 was derived with only death as a competing risk^27^.

**Figure 3:**
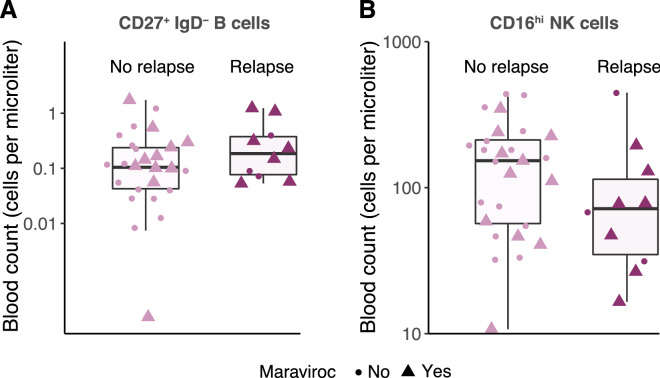
Blood counts (cells/$\mu$l) of CD27$^+$ $\hbox {IgD}^-$ memory B cells (**A**) and CD16$^\text {hi}$ NK cells (**B**) in patients, stratified by relapse and maraviroc treatment.

An association between a variable and the CIF could either be explained by direct effect of that variable, or arise because the variable is associated with an opposing change in the risk of occurrence of a competing event^42^. In our analysis, this ambiguity could be found in the association of higher numbers of effector memory (CD27$^-$CD28$^-$) CD8$^+$ T cells with a higher probability of cGVHD; this could be an indirect effect, because this cell subpopulation is associated with a lower instantaneous risk of relapse. On the other hand, the CSH and SH analyses are consistent regarding the effect of maraviroc in reducing the cumulative incidence of aGVHD24, as well as CD27$^+$ $\hbox {IgD}^-$ memory B cells and increased donor age in increasing the cumulative incidence of relapse and CD16$^\text {hi}$ NK cells in decreasing it. For that reason, these variables can be interpreted as directly influencing the event probabilities^33^. Absolute frequencies of the CD27$^+$ $\hbox {IgD}^-$ memory B and CD16$^\text {hi}$ NK cell populations in serum in the different groups are shown in Fig. 3.

## Discussion

We show here that variable selection methods adapted to the context of high dimensional data and a competing risk model allow us to identify variables associated with the clinical outcome of patients following allo-HCT. Both statistical methods we considered revealed an association of relapse with NK and B cell populations, suggesting that these subsets could be investigated as prognostic factors. We also found associations between the incidence of acute GVHD grade 2–4 and several subpopulations expressing the chemokine receptor CCR5. In summary, this approach is valuable for identifying variables associated with clinical outcome in an unbiased way, exploiting the richness of information provided by gated flow cytometry data. However, here we analyzed a small sample of patients as a proof-of-concept, and the predictive potential of the selected cell subpopulations should be verified using bigger datasets.

The statistical models used in this study also come with strong assumptions that could limit the analysis. In particular, the Cox model assumes that the contribution of each variable to the logarithm of the hazard is linear, but the relationship may be more complex. Moreover, the effect of each variable is adjusted on the others, but we did not explore interactions between covariates: including these would dramatically increase the number of variables in the initial model and would make selection more challenging. In an attempt to address these issues we also explored random forests, which require no assumptions regarding the form of the relationships between the variables and the outcome. However, likely due our relatively small sample of patients, we were not able to obtain any conclusive results with this method. Moreover, the interpretation of results obtained with random forests is harder to express in clinical terms than the one provided by the Cox models, and random forests have been found to perform similarly to the likelihood boosting approach in term of predictive performance^24^.

Additionally, the numbers of cells in the studied subpopulations are very likely correlated. The elastic-net method was preferred here in the case of the cause-specific hazard model, as it has been shown to perform better than the lasso method in the case of correlated covariates. However, another way of handling the specific structure of the data generated by flow cytometry would be to consider predefined groups of cell subpopulations, as is commonly done with groups of genes^43^ and to apply the extended methods of group-lasso^44^, sparse group-lasso^45,46^ or even random forests with grouped variable importance^47^.

Here, we focused on identifying immunological variables measured 30 days after allo-HCT that were associated with three clinical outcomes, using a competing risks model. However, patients can experience multiple events, and to deal with this the analysis could be extended with a multistate model^48,49^. Such models yield the transition hazards between each pair of clinical states *i* and *j*, and have already been applied within the setting of allo-HCT^50–52^, although without the variable selection methods we present here. The transitions are typically between aGVHD24, relapse, cGVHD, and/or infectious disease, that can occur sequentially before the end states of of recovery, death, or a new transplant. However, we were not able to implement a multistate model here, for two reasons. First, a number of medical interventions such as DLI can modify the clinical trajectory followed by a patient. We assumed that the immunophenotyping data available 30 days after the allo-HCT would not be predictive of clinical outcome after a medical intervention performed sometimes months after the allo-HCT. Second, our sample was too small to estimate the effect of the variables on all possible transition rates between clinical states.

After identifying cell subpopulations associated with clinical outcome, a natural extension would be to examine whether their subsequent dynamics within an individual are predictive of the time to a clinical event. This type of analysis can be realized using joint models^53^, in which an underlying random effects structure links the survival model (time to event of interest) and the model describing the time-variation of the relevant variables. This approach allows for individual-specific predictions^54^.

The statistical methods presented in this article allow an unbiased identification of cell phenotypes associated with clinical outcomes following allo-HCT. However, another source of bias arises from the data itself. Typically, in flow cytometric data cell subsets are defined by manual gating, which introduces a potential bias; the gating strategy is fixed in advance, and only the subpopulations of cells assumed to be biologically relevant are measured, as the total number of marker combinations cannot reasonably be explored by hand. A non-parametric method for unbiased cell population discovery, FAUST (Full Annotation Using Shape-constrained Trees) has recently been developed and applied to cancer immmunotherapy clinical trials^55^. This approach could be used to discover new cell subpopulations associated with different clinical outcomes in the framework of allo-HCT.

Our study shows the relevance of sophisticated statistical methods to analyze single-cell data in the framework of allo-HCT to identify immunological variables predictive of clinical outcomes. Such analyses may boost our understanding of the mechanisms underpinning these outcomes.

## Methods

### Data

We analyzed data from a subset of patients who participated in a phase 1-2 clinical trial (NCT0094875) evaluating the safety and efficacy of adding a CCR5 blockage (maraviroc) to standard GVHD prophylaxis (tacrolimus, methotrexate) in reduced-intensity allo-HCT recipients and controls who were contemporary patients with similar characteristics that were treated with standard of care transplant. All patients were recruited between 2009 and 2013^26,27^. For all 37 patients included in the analysis, we had access to the following data: 13 clinical characteristics (recipient age, donor age, sex of donor and recipient, gender match, donor and recipient cytomegalovirus status, diagnosis, matching status, treatment, time to neutrophil engraftment, time to platelet engraftment, number of infections before day 100), time and type of event following HCT (aGVHD24, relapse, cGVHD, DLI, death), and flow cytometric immunophenotyping of samples taken 30 days after allo-HCT (82 cell subpopulations; see ref. ^27^ for details). We focused on identifying variables associated with the time to first event after allo-HCT, censored by follow-up or DLI.

Clinical trial patients and contemporary control patients signed informed consent for the collection and analysis of blood samples on protocols that were performed according to relevant guidelines and approved by the institutional review board at the University of Pennsylvania. The analysis of deidentified data presented here was approved by the institutional review board at Columbia University. All data used for these analyses are provided in Supplementary Data S1 online.

### Competing risks: general framework

After allo-HCT, patients can experience a number of different events. The appropriate statistical framework is a competing risks model^48^, which allows one to estimate the probability of occurrence of each event (by accounting for the possibility of the others occurring). Assuming that *K* different events can occur, we define ${\tilde{T}}_k$ to be the time to event *k* and $k=1 \dots K$ the index variable indicating which event happens first. If no censoring occurs, we observe $T=\min \{{\tilde{T}}_k\}$ and *k*; otherwise the observation is the censoring time *C*. In our case, events after allo-HCT are relapse, aGVHD24, or cGVHD. We censored the observations by the time to DLI, rather than death; none of the patients died without experiencing one of the three clinical events. We focused on the associations between cell subpopulations 30 days after allo-HCT and the first clinical outcome experienced.

The effect of covariates can be assessed on the two following quantities of interest:

- The cause-specific hazard (CSH), which is the instantaneous rate of occurrence of a given event *k* among the patients still event-free. It is the hazard of experiencing event *k* in the presence of the other events:
  1 $$\begin{aligned} h_k(t) = \lim _{\Delta t \rightarrow 0} \frac{P(t\le T<t+\Delta t, \epsilon =k|T \ge t)}{\Delta t} \end{aligned}$$
- The cumulative incidence function (CIF), corresponding to the probability of occurrence of a given event *k* by time *t*. It is the expected proportion of patients that have experienced event *k* by a time *t*:
  2 $$\begin{aligned} I_k(t)= Pr(T\le t,\epsilon =k) = \int _{0}^{t} h_k(u)S(u)du \end{aligned}$$

An important point here is that the effect of a variable on the CSH can differ from its effect on the CIF. Indeed, the survival function *S*(*u*) at the core of the definition of the CIF in equation (2) is the probability of not having experienced any event by time *u*: it therefore depends on the CSHs of all events.

### Selection methods in the competing risks framework

All analyses were performed with R version 3.6.1^56^. Specific packages are referenced below.

#### Cause-specific hazard model

The CSH is modeled by using a Cox model^34^, which can be specified as:

3 $$\begin{aligned} h_k(t|Z) = h_{k0}(t)\exp (\beta _k^T Z) \end{aligned}$$

for $k=1 \dots K$ with $\beta _k$ the vector of coefficients associated with the vector of covariates Z$= (Z_1, \dots Z_m)$, $\hbox {HR}_{k,p}=\exp (\beta _{k,p})$ the hazard ratio (HR) corresponding to covariate $Z_p$ and $h_{k0}(t)$ the baseline hazard for event *k*. The cause-specific hazard model (CSHM) corresponds to regular Cox model for one event at a time, by treating all other events as censored. When the number *p* of covariates is much high than the number *n* of individuals, the classical methods of estimation and selection (e.g., backward/forward selection) perform poorly. In this case, we realize the variable selection by using regularization methods already existing for regression models, including the Cox model. In particular, we focused on the Elastic Net (EN) method^35^, which combines the LASSO^57^ and Ridge^58^ penalizations: these methods rely on adding a penalty on the non-zero coefficients, which shrink them toward zero. In practice, if we note $L_k(\beta _k)$ the partial log likelihood for event $k=1..K$, we estimate $\hat{\beta _k}_{EN}$ as:

4 $$\begin{aligned} \hat{\beta _k}_{EN} = argmax_{\beta _k \in {\mathbf {R}}^p} \Big [ L(\beta _k) - \alpha \lambda \sum _{j=1}^p |\beta _{k,j}| - (1-\alpha ) \lambda \sum _{j=1}^p \beta _{k,j}^2 \Big ] \end{aligned}$$

where $\lambda$ is the penalization penalty, usually determined by cross-validation, and $\alpha$ is the mixing parameter between Ridge ($\alpha =0$) and LASSO ($\alpha =1$). The EN method has been shown to perform better than the LASSO when the covariates are strongly correlated^35^. Analyses were performed using the function *cv.glmnet* from the *glmnet* R package^59^.

#### Fine and Gray subdistribution hazard model

The cumulative incidence functions (CIFs) for the three competing events were computed and plotted using the *cuminc* function from the *cmprsk* R package^60^. To estimate the effect of covariates on each CIF, we applied the Fine and Gray model, which relates the subdistribution hazard for event *k* (${\overline{h}}_k(t)$) to the CIF ($I_k(t)$) with^37^:

5 $$\begin{aligned} {\overline{h}}_k(t) = - \frac{d \log (1 - I_k(t))}{dt}. \end{aligned}$$

One then assumes that the influence of covariates on this subdistribution hazard is described with a Cox model:

6 $$\begin{aligned} {\overline{h}}_k(t|Z) = {\overline{h}}_{k0}(t)\exp ({\overline{\beta }}_k^T Z). \end{aligned}$$

A covariate’s estimated effect on the subdistribution hazard can then be related directly to the CIF. We performed variable selection on the subdistribution hazard using a likelihood-based boosting approach, first developed on survival models^61^ and later extended to the competing risks framework^23^. One feature of this approach is that it does not require variance estimation, which can be problematic in high-dimensional settings. The estimation of the vector of parameters relies on updating its coefficients one-by-one over the course of number of so-called “boosting” steps. At each step, the minimization of a loss function determines which element of the parameter vector is updated. The loss function is based on the partial likelihood of the Cox model. This method has been applied in other settings, such as bladder cancer^62^ and prostate cancer^63^. Our analyses were performed using the function *CoxBoost* from the *CoxBoost* R package^64^.

#### Random forests

The two methods described above are based on the Cox model, which comes at the price of assuming proportional hazards and linearity in the contributions of variables to the outcome. Fully non-parametric methods can be used to analyze the data without making any assumptions regarding the form of the relation between the covariates and the outcome. In particular, random forests^65^, a tree-based approach, have been very popular in classification and regression problems in high-dimensional settings. In this approach one aggregates a number of trees that are grown on a bootstrap sample of the data and by randomly sampling a subset of variables for the splitting of each node. Optimization of a given criterion allows the splitting of the data at each node. Random forests have been extended to the case of survival data and competing risk frameworks^25^, by modifying the splitting rules used and the quantities of interest that are estimated in each terminal node. We tested two survival splitting rules. One was based on the generalized log-rank test for event *k*, which tests the equality of the cause-specific hazard function in the left and right nodes. In this case we ranked the variables based on variable importance^66^. This rule is useful for determining variables affecting the CSH. The other rule was based on Gray’s test, which compares the subdistribution hazard functions and allows selection of variables on the basis of their influence on the CIF. For this rule, we ranked the variables based on their minimum depth, which is not event-specific. We performed the analyses using the functions *rfsrc*, *opt.rf* (which optimizes the parameters of the random forest), *vimp* (to compute the variable importance) and *max.subtree* (to obtain the minimal depth of variables) from the *randomForestSRC * R package^67^.

#### Pipeline for variable selection

Subpopulation numbers were log-transformed, using a threshold of half the minimum number observed in the data. High variance in the selection is already handled in random forests by “bagging” (bootstrap aggregating) a given number of trees, trained on bootstrap samples of the data and randomly sampling a new set of variables for the splitting of each node. We used the following pipeline for the elastic net on the CSHM and the boosting on the SH. First, we performed variable selection for each competing event (relapse, aGVHD24, cGVHD) on 500 bootstrap samples to ensure robustness of the selection. Then, based on the selected variables for all competing events, we ran a final backward/forward selection (based on the Bayesian Information Criterion) to determine the best model and estimate the hazard ratios associated with the selected variable for each event. This selection was made with the function *crrstep* from the *crrstep* R package^68^. All models were adjusted on treatment with maraviroc, and if any variable selected in the final model was a subpopulation expressing the chemokine receptor CCR5, interactions between that variable and the treatment variable were tested. Finally, we checked the validity of the model assumptions, and in particular the proportional hazards for Cox model, with the function *cox.zph* from the *survival* R package^69^. Validation was performed by comparing a model’s time-dependent AUC to that obtained with a model containing only the maraviroc treatment variable: this was done by cross-validation using the function *Score* from the *riskRegression* R package^70^. Only the models for aGVHD24 and relapse were considered for validation, due to the small number of patients experiencing cGVHD as a first outcome.

## Supplementary Information

Supplementary Information 1.

Supplementary Information 2.

Supplementary Information 3.
