# Supplementary material for: Variable selection methods for predicting clinical outcomes following allogeneic hematopoietic cell transplantation
Source: Sci Rep. 2021 Feb 5;11:3230. doi: 10.1038/s41598-021-82562-0 (PMC7865009; doi:10.1038/s41598-021-82562-0)
Supplement: Supplementary file 3 — Supplementary Information 3. [file 41598_2021_82562_MOESM3_ESM.pdf]

Variable selection methods for predicting clinical outcomes following allogeneic hematopoietic cell transplantation: supplementary information

Chloe Pasin, Ryan H. Moy, Ran Reshef, Andrew J. Yates

Supplementary Information

Supplementary Text S1

List of variables

Naive, EMRA, EM and CM T cell subsets were defined using CD45RO and CCR7.

1. Monocytes (CD14+)
2. NK cells
3. NK CD38+
4. NK CCR5+
5. NK CD16hi
6. NK CD16hi CCR5+
7. NK CD16lo
8. NK CD16lo CCR5+
9. NKT cells
10. NKT CD38+
11. NKT CCR5+
12. B cell CD27+
13. B cell IgD+
14. B cell IgM+
15. B cell CD27- IgD+
16. B cell CD27- IgM+
17. B cell CD27+IgD+
18. B cell CD27+ IgM+
19. B cell CD27+ IgD-
20. B cell CD27+ IgM-
21. B cell CD27- IgD-
22. B cell CD27- IgM-
23. B cells
24. B cell CD38+
25. CD8+ T cells
26. CD8+ CD38+
27. CD8+ Naive CCR5+
28. CD8+ Naive
29. CD8+ EMRA CD27- CD28-
30. CD8+ EMRA CD27+ CD28-
31. CD8+ EMRA CD27+ CD28+

32. CD8+ EMRA CD27- CD28+
33. CD8+ EMRA CCR5+
34. CD8+ EMRA
35. CD8+ EM CD27-CD28-
36. CD8+ EM CD27+ CD28-
37. CD8+ EM CD27+ CD28+
38. CD8+ EM CD27- CD28+
39. CD8+ EM CCR5+
40. CD8+ EM
41. CD8+ CM CCR5+
42. CD8+ CM CD28+ CD27+
43. CD8+ CCR5+
44. CD8+ CD28- CD57+
45. CD8+ CD28+ CD57-
46. CD8+ CD28- CD57-
47. CD8+ CD25+
48. CD8+ CTLA4+
49. CD8+ CM
50. CD4+ T cell
51. CD4+ CD38+
52. CD4+ CCR5+
53. CD4+ CM CD27+ CD28+
54. CD4+ CM CCR5+
55. CD4+ EM
56. CD4+ EM CCR5+
57. CD4+ EM CD27- CD28+
58. CD4+ EM CD27+ CD28+
59. CD4+ EM CD27+ CD28-
60. CD4+ EM CD27- CD28-
61. CD4+ EMRA
62. CD4+ EMRA CCR5+
63. CD4+ EMRA CD27- CD28+
64. CD4+ EMRA CD27+ CD28+
65. CD4+ EMRA CD27+ CD28-
66. CD4+ EMRA CD27- CD28-

67. CD4+ Naive
68. CD4+ Naive CCR5+
69. CD4+ Naive CD31+
70. CD4+ CD28- CD57+
71. CD4+ CD28+ CD57-
72. CD4+ CD28- CD57-
73. CD4+ CD25+
74. CD4+ CTLA4+
75. CD4+ CM
76. CD4+ FoxP3+ (Treg)
77. Treg CCR5+
78. Treg CD45RA+
79. Treg CD45RA+ CCR5+
80. Treg CD25+
81. Treg CTLA4+
82. Absolute lymphocyte count (ALC)

Clinical markers:

1. Age recipient
2. Age donor
3. CMV recipient
4. CMV donor
5. Sex recipient
6. Sex donor
7. Sex match
8. Donor type
9. Diagnosis
10. Maraviroc
11. Time to neutrophil engraftment
12. Time to platelet engraftment
13. Number of infections before day 100

Supplementary Figure S1

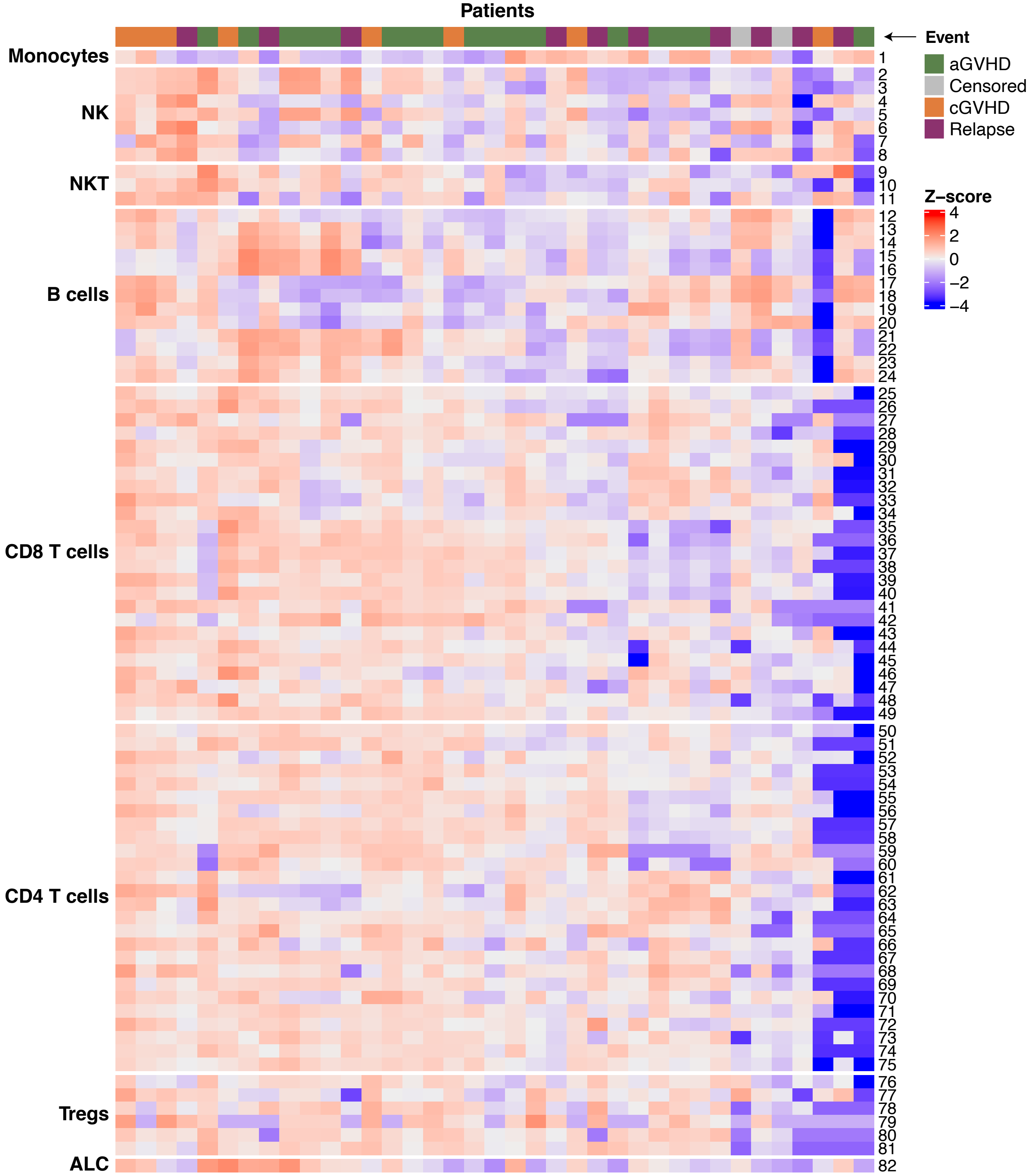

Figure S1: Z-scores of cell subpopulation frequencies across all patients (columns). Z-scores were computed by normalizing each cell subpopulation frequencies, using the formula $Z = (X - \overline{X})/s$, $\overline{X}$ being the mean value in the sample and s the standard deviation. Line numbers correspond to the subsets listed above. Figure generated in R, version 3.6.1 (R Foundation for Statistical Computing, 2020, `https://www.R-project.org/`).
